# Palliative care communication between patients with intellectual disabilities and hospice staff: a Conversation Analysis pilot study protocol

**DOI:** 10.1136/bmjopen-2025-101622

**Published:** 2025-06-26

**Authors:** Andrea Bruun, Irene Tuffrey-Wijne

**Affiliations:** 1 Department of Public Health, Children’s, Learning Disability and Mental Health Nursing, Kingston University London, Kingston upon Thames, UK

**Keywords:** PALLIATIVE CARE, Adult palliative care, QUALITATIVE RESEARCH

## Abstract

**Abstract:**

**Introduction:**

Communication challenges are among the main barriers for people with intellectual disabilities in accessing palliative care. They include inadequate skills among staff and difficulties with confirming understanding and around the presentation and assessment of symptoms. In-depth analysis of interactions between people with an intellectual disability and staff may shed light on these communicative challenges as well as facilitators. However, no studies have closely analysed the interactions between people with an intellectual disability and professionals within palliative care settings.

**Methods and analysis:**

This protocol describes a pilot study assessing the feasibility and acceptability of conducting a Conversation Analysis study involving video-recording palliative care conversations between people with intellectual disabilities and professionals.

Three conversations between patients with an intellectual disability, their companions and palliative care staff will be video recorded in a UK hospice. Recordings will be transcribed and analysed using Conversation Analysis. Communication phenomena of interest and worth further exploration will be identified in collaboration with key stakeholders.

**Ethics and dissemination:**

The study received a favourable opinion by a UK research ethics committee in February 2025. All participants must provide informed consent to take part in the study. It will be carefully assessed that potential participants with an intellectual disability have capacity to consent to take part. Accessible study information materials for participants with an intellectual disability are available (ie, easy-read and video).

Study findings will be disseminated in academic papers and conference presentations. Progress and findings will also be shared via social media and with relevant groups of people with intellectual disabilities, family carers, service providers and academics.

**STRENGTHS AND LIMITATIONS OF THIS STUDY**

- Conversation Analysis provides in-depth insights into the communication (both verbal and non-verbal) between patients with an intellectual disability, their companions and staff.
- Researchers with an intellectual disability have ensured study information materials are accessible and inclusive.
- Conversation Analysis data sessions with experts and key stakeholders will ensure the relevance of the future project, which the pilot study will inform.
- The small sample size may lack overall representability regarding the feasibility of the study; particularly regarding people who lack capacity to provide informed consent to take part in the study.

## Introduction

 People with intellectual disabilities experience marked health inequities and are less likely to access skilled end-of-life support, including specialist palliative care.^1^ Addressing these inequalities in accessing palliative care for people with intellectual disabilities has been labelled an urgent priority globally.^2^ Challenges around communication have been identified among the main barriers to providing appropriate palliative care as it affects all aspects of care provision.^1^ Communication is also included as a priority in the consensus norms for palliative care of people with intellectual disabilities in Europe.^1^

Specific communication barriers include inadequate communication skills among staff, complex medical language, assumed lack of capacity of the person with an intellectual disability, difficulties with confirming understanding, ascertaining information, establishing wishes^2^ and difficulties around the presentation and assessment of pain and other symptoms.^3^ Facilitators include a person-centred approach and tools to assess symptoms^2^ as well as using communication aids (eg, pictures and objects of reference), taking time and using simple language.^4^

Most studies within the field have explored communication by eliciting participants’ (mainly staff) experiences with and perspectives of communication through interviews or focus groups.^5 6^ There is a prominent lack of studies exploring palliative care communication from the perspective of people with intellectual disabilities themselves.^7^ Communication itself may be a barrier to research participation where some methodologies require that the person can express themselves in an understandable way to the researcher. This may lead to exclusion of people with certain communication impairments and those with more severe and profound intellectual disabilities.^8^ Subjective retrospective participant accounts of communication do also not offer the level of detail needed to properly understand how these interactions are carried out in practice.

Direct observations or analysis of recorded interactions provide more detailed insights into the intricacies of the interaction between the person with an intellectual disability and palliative care staff. These methods also enable people with severe and profound intellectual disabilities to take part in research.^9 10^ There is a body of research involving in-depth analyses of recorded interactions within palliative care settings.^11 12^ The need for future research exploring palliative care interactions involving overlooked participant groups and people in underserved and minoritised communities have been highlighted.^11^ In-depth analyses of interactions with people with an intellectual disability in other healthcare settings have been conducted.^13^ However, no studies have closely analysed the interactions between people with an intellectual disability and professionals within palliative care settings. In-depth analysis of such interactions can gain insights into specific communicative challenges and facilitators, which can be used to further inform and develop recommendations and guidelines.

This pilot study takes the first step in addressing the prominent gap in the literature by exploring palliative care communication through analysis of video-recorded interactions between people with intellectual disabilities, their companions and health and social care staff in a hospice.

### Study aims and objectives

The overall aim of this project is to assess the feasibility and acceptability of conducting a Conversation Analysis study involving video-recording palliative care conversations between people with intellectual disabilities and professionals.

The objectives are:

- to assess recruitment, data collection tools, and study procedures of video-recording palliative care consultations between people with intellectual disabilities and professionals in a UK hospice.
- to conduct preliminary analyses of video-recordings of palliative care consultations between people with intellectual disabilities and professionals to identify communication phenomena of interest.

The pilot study findings will inform and support a future research funding application using Conversation Analysis aiming at developing recommendations and guidelines for communication between professionals and people with intellectual disabilities within palliative care settings.

## Methods and analysis

This is a small-scale Conversation Analysis pilot study, involving video-recording palliative care interactions between people with intellectual disabilities, their companions and professionals in a hospice. The study period spans from March to July 2025.

### Setting

The study will take place in a hospice located in the UK. In the UK, hospices provide free nursing and medical care for anyone with a terminal illness or life-limiting condition as well as emotional, psychological, spiritual and practical support.^14^ Hospice care can be provided at any stage of a person’s illness or condition, not only at the end of their lives.^15^ Most hospices provide a range of services such as inpatient care (ie, short-term admissions and end-of-life care admissions in the last weeks or days of life); outpatient care (eg, support groups, exercise programmes, creative and complementary therapies, counselling and spiritual support); hospice care at home and support for patients’ families and friends.^14^

The collaborating hospice in this study offers both palliative care inpatient and outpatient as well as day services. Their services include specialist clinical care, supportive care and creative and complementary therapies. The hospice collaborators have experience with caring for people with intellectual disabilities and with being involved in research.

### Sample

Study participants are adult patients with an intellectual disability, and their companions, who are having conversations with health and social care professionals in the collaborating hospice.

We will recruit three study participants who meet the following inclusion criteria:

- Has an intellectual disability.
- Aged 18 or over.
- Has capacity to provide informed consent to take part in the study.
- In receipt of services and/or support from the collaborating hospice.
- Has had, and will have more, conversations with a health or social care professional from the collaborating hospice.
- If they have a companion who is part of the conversation, the companion agrees to be part of the study.
- The health and social care professional involved in the conversation agrees to be part of the study.

Each person taking part in the consultation (eg, person with an intellectual disability, their companion and professional) will be recruited as participants, totalling between 6 and 15 participants. The total number of participants accounts for the person potentially having two companions present (eg, a parent and a sibling) and should the conversation involve two professionals (eg, a doctor and a nurse). The minimum number of participants is six if all three conversations are one-to-ones between the person with an intellectual disability and the hospice professional.

The sample size is kept small to account for the short study period permitted by the funder; a total of 4 months. This only allows 2 months for data collection, including participant recruitment. Studying communication within underserved populations such as intellectual disability may involve recruitment challenges. The researchers experienced this in their latest Conversation Analysis study of funeral planning sessions with people with intellectual disabilities and their support workers.^16^ People with an intellectual disability also represent a minority of the general population, and there are no hospice services specifically for people with an intellectual disability in the UK. Despite the hospice collaborator having experience with caring for patients with intellectual disabilities, their main patient group is people without intellectual disabilities. For this reason, the study involves what has been described as time-intensive and resource-intensive opportunistic sampling where eligible people happen to ‘pop up’ in a service.^13^ Additional challenges also include that people represent an even smaller minority of the population that access healthcare,^13^ and, as described previously, people with intellectual disabilities are less likely to access palliative care.^1^

### Participant recruitment and consent

The hospice collaborators will help identify potential participants, inform them about the study and distribute study information, including easy-read materials and an information video. Potential participants will have opportunity to ask questions and be given time to consider their involvement in the research. The researcher will also be available to provide further information and support people with an intellectual disability in understanding the research study information and deciding whether to participate. Informed consent will be obtained by the researcher.

All research participants will provide informed consent using either a written or verbal consent form, including an accessible easy-read consent form for people with an intellectual disability. If using the verbal consent form, the researcher will read out each statement and the participant will be asked to indicate whether they agree to each point. The researcher will sign the form to indicate the participant has agreed. Verbal consent is appropriate both for people with intellectual disabilities who struggle to read or write, and for people who may wish to give consent over the telephone or using video-calling platforms.

Anonymised details of potential participants declining to participate in the study will be recorded as field notes and treated as data. This data will capture information about the potential participant, the circumstances and reasons for declining to participate in the research as this may contribute to understanding the degree to which participants accept the study and its procedures.

After obtaining formal written or verbal consent, we will follow an ongoing consensual process (‘process consent’).^17^ If the participant communicates or demonstrates any unwillingness to participate in the research before or during the conversation (eg, does not want to be video-recorded), then the conversation will not be recorded or, (if the recording has already started), the recording will be stopped. Furthermore, if the camera is disrupting the conversation (eg, the person is not engaging in the conversation as they normally would), then the camera will be turned off and removed. The researcher will ask all participants to consider and pay attention to how the person may express that the camera is disrupting the conversation. In this case, they will be asked to let the researcher know, so the recording can be discontinued. Both scenarios will be considered as the person withdrawing their consent to have their conversation recorded, and they will be withdrawn from the study.

Special consideration will be given to the patient’s condition on the day. If they are too ill, tired or sedated and are deemed to have lost capacity to consent, the recording will not take place.

#### Capacity to consent

Capacity to consent will be assessed in all study participants, with extra care taken for participants with intellectual disabilities. Capacity to consent will be assumed (as per the Mental Capacity Act) unless there is reason to suggest the person does not have capacity. If the research team has any doubts about a person’s capacity to consent, they will seek advice from someone who knows the person well. The assessment of the potential participant’s capacity will be made by the research team based on whether the person is able to:

- Understand the nature of this part of the study.
- Understand what participating in the study involves.
- Understand the advantages and disadvantages of taking part in the study.
- Understand that they can say ‘no’ to taking part, or withdraw at any time.
- Retain the information for long enough to make a decision.
- Make this particular decision.
- Make a free choice.

If the researcher assesses that the person does not have capacity to make the decision, the person cannot take part in the research and will be excluded from the study.

### Data collection

We will collect demographic information from all participants, which includes age, gender, nationality and ethnicity. Job titles and the number of years of work experience from staff and paid carer companions; family companions’ relationship with the person with an intellectual disability and the health status of people with intellectual disabilities will also be recorded.

The researcher will assist with recording the conversations, using high-quality video-recording equipment. The recording equipment will be set up in the room before the conversation begins. The researcher will not be present during the conversation to minimise researcher influence on the interactions.

Data collection will take place between March and May 2025.

### Data analysis

Video-recordings will be analysed using Conversation Analysis. This is a method that investigates how people interact with each other through close analysis of how natural conversations unfold.^18^ Conversation Analysis examines not just what is said, but also how it is said (eg, intonation, pauses and non-verbal embodied communication practices). This is particularly important when examining communication with people with intellectual disabilities who often use alternative ways of communicating that would be missed if only focusing on the spoken word. Conversation Analysis also focuses on all parties in the interaction (ie, both the person with an intellectual disability, their companion and the staff member) to understand how what one person communicates impacts the other’s response. It is increasingly seen as the gold standard for analysing the structure and functioning of health and social care communication and has shown promising results when used to design and evaluate interventions aimed at improving communication in practice.^19 20^

Recordings will be transcribed using standard Conversation Analysis conventions^21^ and analysed using the conversation analytic method. This will involve the identification of sequences—series of communication turns—of interest. These sequences will be examined to understand how the interactions are carried out in practice and shed light on potential facilitators and barriers. For example, which strategies are used by people with intellectual disabilities, their companions and staff; how are topics and/or concerns raised (and by whom), how is information shared; how are decisions made and how are physical resources (eg, easy-read materials and pictures) brought into the interaction. These practices will then be examined with reference to where they occur in the interaction, and how they are designed and organised.

Short sections will be played in confidential data sessions with external conversation analysts with palliative care and intellectual disability research expertise. Attendees at data sessions will be invited to give their observations about the interaction, allowing new interpretations of the data to be discussed and providing checks on analyses already conducted.^22^ Data sessions with people with intellectual disabilities, companions and staff may also be held. When organised appropriately, data sessions enable input from participants with no formal conversation analytic training and allow equal contributions from participants with a range of expertise and experience.^23^

Based on the analyses, communication phenomena of interest and worth further exploration in the future research funding application will be identified in collaboration with key stakeholders. Potential communication phenomena include those listed above (eg, information-sharing, decision-making, use of physical resources). Based on previous Conversation Analysis research in the fields of palliative care and intellectual disability, other potential communication phenomena include discussion of illness progression and the end-of-life,^24^ pain assessment,^25^ how companions speak on behalf of the patient^26 27^ and how to deal with undecipherable talk.^28^

### Patient and public involvement

The research team has worked actively with professionals, families and people with intellectual disabilities in previous studies around palliative care and death and dying; this has included discussions around the challenges and the need for investigating palliative care communication. People with intellectual disabilities have expressed concerns about not being able to understand what happens when they are ill and admitted to hospice or hospital, and the fear of staff not understanding them and what they want. They consistently express their distress at staff using ‘too much jargon’. Families, particularly of people with severe and profound intellectual disabilities, are worried that their loved ones will not get the right care if they are not around. Staff also often express a need for more specific communication guidance.

The researchers are part of a wider inclusive team at Kingston University London that includes four researchers with an intellectual disability, where they benefit from continuous feedback. Researchers with intellectual disabilities in this team also lead the Yellow Tulip group, a monthly online group that discusses inclusive research, including research about death and dying for people with intellectual disabilities. People with intellectual disabilities and their supporters are regular members of this online group. This group has advised on how to create accessible study information materials, including easy-read forms and videos.

The researchers are both working on a new study titled ‘Developing effective service models for Adult Palliative and end of life care for People with a LEarning disability (DAPPLE)’.^29^ Procedures and findings from the pilot study will be presented and discussed in the projects monthly co-production group of people with intellectual disabilities.

People with an intellectual disability, their companions and staff will be part in the data analysis by identifying the communication phenomena that warrant further exploration in the forthcoming research funding application. They will also help with disseminating the study findings.

This is a small-scale pilot study that will hopefully lead to a future research funding application, which will involve a researcher with an intellectual disability working alongside the Principal Investigator; using Experience Based Co-Design methods to develop communication recommendations; and a Research Advisory Group with people with intellectual disabilities, families, staff and policy-makers.

## Ethics and dissemination

The study received ethical approval from West Midlands—Coventry & Warwickshire Research Ethics Committee (25/WM/0012) on 20 February 2025.

There are important ethical considerations with regards to informed consent. It is crucial that people with intellectual disabilities are given the opportunity to take part in research. Analysing video-recordings of real-life interactions is one of the few ways in which their perspectives and experiences can be included in research. We will therefore assume participants have capacity to consent (as per the Mental Capacity Act) unless there is reason to suggest otherwise (see the Capacity to consent section). The research team is highly experienced in conducting research of this kind with vulnerable groups and knowledgeable about the ethical principles underpinning informed consent and the Mental Capacity Act (see the *Capacity to consent* section for details).

The researchers are part of a wider team at Kingston University London that includes researchers with an intellectual disability. They have been consulted to ensure that study information materials are in accessible formats, including easy-read and videos. If needed, information will be explained with the support of someone who knows the participant well.

It is also important to consider the management of distress around a sensitive research topic. All participants will be given the details of a named person within the hospice, as well as a member of the research team, whom they can contact afterwards, if they wish to talk further about their participation in the study or debrief. They will also be able to advise participants on where they can access further support if needed.

The study will identify barriers and enablers to recruitment, data collection tools and study procedures of video-recording palliative care conversations between people with intellectual disabilities, their companions and professionals. The findings will be used to inform the future research funding application.

A small dataset of three recordings will be created and used to identify communication phenomena of interest. These findings will also be used to inform the future project. A network of key stakeholders will be developed, who will be invited to collaborate on the future project.

The preliminary analyses will be further developed and turned into papers and conference presentations. This includes the study protocol in question, which can be referenced and used as a springboard when writing the future research funding application. Progress and findings will also be shared via social media. We will present our findings to relevant groups including people with intellectual disabilities, family carers, self-advocacy and carer groups, service providers and academics.
