# Supplementary material for: Palliative care communication between patients with intellectual disabilities and hospice staff: a Conversation Analysis pilot study protocol
Source: BMJ Open. 2025 Jun 26;15(6):e101622. doi: 10.1136/bmjopen-2025-101622 (PMC12207180; doi:10.1136/bmjopen-2025-101622)
Supplement: Supplementary file 1 [file bmjopen-2025-101622.reviewer_comments.pdf]

PEER REVIEW HISTORY

BMJ Open publishes all reviews undertaken for accepted manuscripts. Reviewers are asked to complete a checklist review form and are provided with free text boxes to elaborate on their assessment. These free text comments are reproduced below.

ARTICLE DETAILS

Title (Provisional)

Palliative care communication between patients with intellectual disabilities and hospice staff: a Conversation Analysis pilot study protocol

Authors

Bruun, Andrea; Tuffrey-Wijne, Irene

VERSION 1 - REVIEW

**Reviewer**	**1**
**Name**	**Lakin, Joshua R**
**Affiliation**	**Brigham and Women's Hospital and Harvard Chan School of Public Health**
**Date**	**06-May-2025**
**COI**	**None**

Thank you for the opportunity to review this manuscript; I very much enjoyed reading it and I believe that the topic is relevant for readers of BMJ Open. The authors describe a protocol for a pilot study employing conversational analysis to assess the communication elements deployed in initial consults for patients in a British hospice setting. The manuscript is clearly written, presents interesting and novel details, and addresses a very important topic relevant to hospice and palliative medicine and more broadly.

Major Concerns and Questions:

-I have no major concerns or questions.

Minor Concerns and Questions:

-In results, page 3 – I'm not quite clear on what the authors mean by "palliative care conversations" as this can include a wide variety of clinical interventions – from early symptom assessment in chronic illness to advance care planning and serious illness conversations all the way to end of life discussions. I think some detail for readers, particularly since this is not a palliative care specific journal, will be helpful to add specificity and clarity. What specific types of conversations will they be aiming to capture and analyze

in this work? It sounds like they will be recording initial consultations, which can be a bit more structured but can still cover a wide range of clinical communication tasks.

-Reading further down, it sounds like this pilot is taking place in a hospice setting. In the United States, those who qualify for hospice are quite sick – required certification from clinicians as having less than 6 months to live. This population sounds quite unique from what would be happening in a palliative care consultation in the United States. It may be different in the UK but more detail around this would be helpful – how sick are these patients? Might this study actually be evaluating pilot data on hospice conversations? Rather than palliative care consultations. This is still great work and this choice of population and setting might affect generalizability and need more clarity in the language – such as the title and framing that this is hospice rather than palliative care – and should be clearly framed for readers in a way we can assess whether it is relevant to different other unique palliative care settings beyond hospice.

-The journal requires dates of the planned study. I may have missed this but I did not see it in the manuscript. I apologize if I did miss it!

Thank you again for the opportunity to review this paper and thank you to the authors for their impressive work. I immensely enjoyed reading this work and have been hungry to see something like this tool for some time. Congratulations on your efforts.

Reviewer 2

Name Heckel, Maria

Affiliation Friedrich-Alexander-Universität Erlangen-Nürnberg, University Hospital Erlangen, Department of Palliative Medicine

Date 14-May-2025

COI None

Thank you very much for your well-structured and comprehensive study protocol. It addresses all relevant ethical considerations. Your study is highly relevant. The proposed study is a pilot study with a small number of participants, which is appropriate given the population investigated. The design allows testing the recruitment, methods and data analysis. I assume you have considered which specific conversation phenomena may arise. I would suggest to details them in the study protocol. Please consider to clarfiy the terminology used to refer to individuals who accompany participants. In some sections the term family is used in others the term companions. Unfortunatly, due to journal policies, I have to reject the study protocol manuscript when your data collection period is in the past and completed (March to April 2025) according to the manuscript.

VERSION 1 - AUTHOR RESPONSE

Reviewer comments	Author response	Page #
**Reviewer 1**		
Thank you for the opportunity to review this manuscript; I very much enjoyed reading it and I believe that the topic is relevant for readers of BMJ Open. The authors describe a protocol for a pilot study employing conversational analysis to assess the communication elements deployed in initial consults for patients in a British hospice setting. The manuscript is clearly written, presents interesting and novel details, and addresses a very important topic relevant to hospice and palliative medicine and more broadly.<br><br>Major Concerns and Questions:<br>-I have no major concerns or questions.<br><br>Minor Concerns and Questions:		
-In results, page 3 – I'm not quite clear on what the authors mean by "palliative care conversations" as this can include a wide variety of clinical interventions – from early symptom assessment in chronic illness to advance care planning and serious illness conversations all the way to end of life discussions. I think some detail for readers, particularly since this is not a palliative care specific journal, will be helpful to add specificity and clarity. What specific types of conversations will they be aiming to capture and analyze in this work? It sounds like they will be recording initial consultations, which can be a bit more structured but can still cover a wide range of clinical communication tasks.<br>-Reading further down, it sounds like this pilot is taking place in a hospice setting. In the United States, those who qualify for hospice are quite sick – required certification from clinicians as having less than 6 months to live. This population sounds quite unique from what would be happening in a palliative care consultation in the United States. It may be different in the UK but more detail around this would be helpful – how sick are these patients? Might this study actually be evaluating pilot data on hospice conversations? Rather than palliative care consultations. This is still great work and this choice of population and setting might affect generalizability and need more clarity in the language – such as the title and framing that this is hospice rather than palliative care – and should	We acknowledge this may be very country specific, and we have now specified what hospice care means in the UK to clarify this. In the UK, hospices provide a wide range of palliative care services, not only end-of-life care. Patients can be involved with and receive palliative care services from hospices for years. In this way, people are not necessarily quite sick, but may simply come in for reviews/check-ins, pampering sessions, short time respite stays, exercise programmes, or complementary therapies. We are including any type of hospice-related conversation that patients may have – not only clinical consultations. For this reason, we have kept the eligibility criteria broad to be able to include a range of different conversations with different types of staff:<br>• In receipt of services and/or support from the collaborating hospice.<br>• Has had, and will have more, conversations with a health or social care professional from the collaborating hospice.<br><br>We acknowledge that readers may want to know straight away that we are focusing on the hospice setting. In the revised version of the manuscript, we have updated the title to include the word "hospice" to clearly indicate this as the palliative care setting.	4; 1

be clearly framed for readers in a way we can assess whether it is relevant to different other unique palliative care settings beyond hospice.		
-The journal requires dates of the planned study. I may have missed this but I did not see it in the manuscript. I apologize if I did miss it!<br><br>Thank you again for the opportunity to review this paper and thank you to the authors for their impressive work. I immensely enjoyed reading this work and have been hungry to see something like this tool for some time. Congratulations on your efforts.	The study dates have been added to the methods section.	4
**Reviewer 2**		
Thank you very much for your well-structured and comprehensive study protocol. It addresses all relevant ethical considerations. Your study is highly relevant. The proposed study is a pilot study with a small number of participants, which is appropriate given the population investigated. The design allows testing the recruitment, methods and data analysis. I assume you have considered which specific conversation phenomena may arise. I would suggest to details them in the study protocol.	We have already listed some of the phenomena, which could be interesting to further explore:<br><br>"For example, which strategies are used by people with intellectual disabilities, their companions, and staff; how are topics and/or concerns raised (and by whom), how is information shared; how are decisions made; and how are physical resources (e.g., easy-read materials and pictures) brought into the interaction."<br><br>We have now listed some further suggestions at the end of this section as well.	7
Please consider to clarfiy the terminology used to refer to individuals who accompany participants. In some sections the term family is used in others the term companions.	We have made this clearer in the revised version of the manuscript.	6
Unfortunatly, due to journal policies, I have to reject the study protocol manuscript when your data collection period is in the past and completed (March to April 2025) according to the manuscript.	Our data collection period was extended to the end of May 2025. We have updated this in the revised version of the protocol. We submitted our paper before having completed data collection and the journal editor has reassured us the following via email:<br><br>"We consider protocols that have not yet begun data analysis at the time of submission to ensure that we are seeing the original plan for the study. As such, there should be no issue with the circumstances of your study and that I am sending the manuscript for peer review now."	4
